# The Work of the Holy Ghost

**Published:** 1887-07-23

**Authors:** 


### Words of Consolation.

[Communications for this column should be addressed, "Chaplain," care of the Editor of THE HOSPITAL, who will gratefully welcome any suggestions or inquiries from matrons, nurses, and the staff generally.]

XLII.—The Work of the Holy Ghost.

"I will not leave you comfortless" (John xiv, 18), means "I will not leave you 'orphans.' Ye shall never feel desolate, nor bereaved of me. I will come to you, although the world seeth me no more." How truly the promise was fulfilled in the first disciples. The world marvelled much at their endurance, but more at their joy in tribulation, which was so conspicuous just at times when ordinary people would have been overcome with sorrow. The spectacle of men and women abounding in joy in the face of imprisonment, cruelty, or violent death, was a problem the heathen could not solve. They could see the results of the Presence: but the Spirit Himself they could neither see nor know. This is still the case under different circumstances. Worldly minds are still found wondering at, if not admiring, the patience, hopefulness, and joy exhibited by good Christians in affliction. They see the happy results, but they do not perceive that there is a supernatural cause; for they neither see the Comforter nor know Him. Many of us who have faith might perhaps give the Holy Ghost more glory than we do when we behold Him sustaining the sick, and lifting them up just when we expect to find them cast down. You see a face lighted up in the midst of tears, or a countenance beaming with cheerfulness and resignation, with the prospect of a painful operation, or when the sentence of recovery being impossible has gone forth from the physician's lips. You are rather surprised. It takes a moment's reflection to tell you that the cause of the patient's happy condition is supernatural. It is because the Comforter is there: there with the soul He has long convinced, and taught, and guided: there with the soul who has listened to His pleading and obeyed: there with one in whom He has lived, producing at the most critical period of life the fulness of the Beatitude, "Blessed are they that mourn, for they shall be comforted" (Matt. v, 4).

There can be nothing more encouraging to the faith of those who have to minister to sick people than these occasional glimpses of the Comforter's work. We may sometimes go a long time without being sure that we have witnessed more than what is a merely earthly determination to endure what cannot be cured. There is certainly a kind of clinging to hope in many worldly minds when afflicted, a sort of "don't give way" attitude, which is not without its consolation; for it is a species of fortitude, and much pleasanter to deal with than moroseness, or despondency. We will call this the confidence of the natural heart. It is something very different from the comfort of the Holy Ghost. The one is a poor, unstable reed, and may break at any time. The other is the real presence and support of a Divine person, bringing with Him a radiant glow and messages of peace, which make the worst tribulation appear small in comparison with the glory to be revealed. The manifestation of this comfort may not be always brilliant, nor indeed always perceptible; nevertheless, when once established in the soul it is usually permanent. "He may abide with you for ever" (John xiv, 16).

(*To be continued.*)

